# The Cytoplasmic Dynein Associated Protein NDE1 Regulates Osteoclastogenesis by Modulating M-CSF and RANKL Signaling Pathways

**DOI:** 10.3390/cells11010013

**Published:** 2021-12-22

**Authors:** Bhaba K. Das, Jyoti Gogoi, Aarthi Kannan, Ling Gao, Weirong Xing, Subburaman Mohan, Haibo Zhao

**Affiliations:** 1 Southern California Institute for Research and Education, Long Beach VA Healthcare System, Long Beach, CA 90822, USA; jyoti.gogoi@va.gov (J.G.); aarthi.kannan@va.gov (A.K.); ling.gao@va.gov (L.G.); haibo.zhao@va.gov (H.Z.); 2 Department of Dermatology, University of California Irvine, Irvine, CA 92697, USA; 3 Musculoskeletal Disease Center, VA Loma Linda Healthcare System, Loma Linda, CA 92357, USA; weirong.xing@va.gov (W.X.); subburaman.mohan@va.gov (S.M.)

**Keywords:** osteoclast, bone resorption, bone remodeling, cytoplasmic dynein, NDE1, NDEL1

## Abstract

Cytoskeleton organization and lysosome secretion play an essential role in osteoclastogenesis and bone resorption. The cytoplasmic dynein is a molecular motor complex that regulates microtubule dynamics and transportation of cargos/organelles, including lysosomes along the microtubules. LIS1, NDE1, and NDEL1 belong to an evolutionary conserved pathway that regulates dynein functions. Disruption of the cytoplasmic dynein complex and deletion of LIS1 in osteoclast precursors arrest osteoclastogenesis. Nonetheless, the role of NDE1 and NDEL1 in osteoclast biology remains elusive. In this study, we found that knocking-down Nde1 expression by lentiviral transduction of specific shRNAs markedly inhibited osteoclastogenesis *in vitro* by attenuating the proliferation, survival, and differentiation of osteoclast precursor cells *via* suppression of signaling pathways downstream of M-CSF and RANKL as well as osteoclast differentiation transcription factor NFATc1. To dissect how NDEL1 regulates osteoclasts and bone homeostasis, we generated *Ndel1* conditional knockout mice in myeloid osteoclast precursors (*Ndel1*^ΔlysM^) by crossing *Ndel1-*floxed mice with *LysM*-Cre mice on C57BL/6J background. The *Ndel1*^ΔlysM^ mice developed normally. The µCT analysis of distal femurs and *in vitro* osteoclast differentiation and functional assays in cultures unveiled the similar bone mass in both trabecular and cortical bone compartments as well as intact osteoclastogenesis, cytoskeleton organization, and bone resorption in *Ndel1*^ΔlysM^ mice and cultures. Therefore, our results reveal a novel role of NDE1 in regulation of osteoclastogenesis and demonstrate that NDEL1 is dispensable for osteoclast differentiation and function.

## 1. Introduction

Maintenance of skeletal integrity and strength is a tightly regulated process of continuous bone remodeling orchestrated by a closely balanced interplay between bone-resorbing osteoclasts and bone-forming osteoblasts [1,2]. Abnormal bone resorption due to increased or decreased osteoclast number/activity is associated with multiple pathological skeleton conditions, including osteoporosis, rheumatoid arthritis, Paget’s disease of bone, and osteopetrosis [3]. Therefore, deciphering the mechanisms that regulate osteoclast differentiation and function will not only lay new insights into osteoclast biology but also significantly improve our understanding and treatment of metabolic bone diseases.

Osteoclasts are multinucleated hematopoietic lineage cells derived from fusion of mononuclear precursors of the monocyte/macrophage family [4,5]. Osteoclastogenesis *in vivo* and *in vitro* is triggered by two key cytokines, M-CSF (macrophage colony-stimulating factor) and RANKL (receptor activator of nuclear factor-kB ligand). M-CSF regulates the proliferation and survival of osteoclast lineage cells by activating the ERK (extracellular signal-regulated kinase) and PI3K/AKT (phosphoinositide-3-kinase/Akt) pathways. RANKL magnifies the MAPKs (mitogen-activated protein kinases), NF-κB, and PI3K/AKT pathways [6,7,8]. RANKL, along with the immunoreceptors and their adaptor proteins, co-stimulate intracellular calcium signaling, which induces the master transcription factor of osteoclast differentiation, NFATc1 (nuclear factor of activated T-cells 1) [9].

Attachment of osteoclasts to bone matrix results in polarization and activation of osteoclasts manifested by extensive cytoskeletal reorganization and lysosome secretion [10,11]. When cultured on a non-mineralized matrix (such as glass coverslips and plastic culture dishes) *in vitro*, the individual podosomes, highly dynamic structures enriched of filamentous actin (F-actin), are organized into a “podosome-belt” encircling the cell periphery [12,13]. When sitting on mineralized bone matrix (dentin or cortical bovine bone slices), the high-density of podosomes form a ring-like structure, so called actin-ring, which tightly seals the resorptive microenvironment in resorbing osteoclasts [14]. Although significant advances have been made in understanding the regulatory mechanisms of actin-cytoskeleton organization in osteoclasts during the past decades, comparatively less is known about the role of microtubules in osteoclast activation and function. Tubulin acetylation is critical for microtubular organization. Suppression of this tubulin posttranslational modification disrupts the formation and stability of podosome-belt and actin-ring in osteoclasts, thus impinging bone resorption [15]. In addition to providing cells with structural integrity, microtubules also act as highways for intracellular transportation of membrane-bound vesicles and non-membrane-bound cargo proteins [16]. This process is powered by ATPase-associated mechanochemical motor proteins of the kinesin or dynein family [17].

The cytoplasmic dynein is a multi-subunit complex comprising heavy chains, intermediate chains, light-intermediate chains, and light chains [18]. In addition, multiple adaptor proteins bind to dynein and regulate its activities, including the dynactin complex, BICD2 (Bicaudal D2), LIS-1 (Lissencephaly-1), NDE1 (Nuclear distribution protein E 1, also known as NudE), NDEL1 (Nde1-like, also known as NudEl) and Tctex-1 (t-complex testis-expressed-1) [19]. Disruption of dynein activity inhibits osteoclastogenesis and bone resorption *in vitro* [20]. Knocking-down Tctex-1 by RNA interference retards bone resorption by blocking Rab3D-mediated vesicular secretion in osteoclasts [21]. On the other hand, the 8-kDa dynein L chain (LC8) negatively regulates osteoclast differentiation and function by suppressing RANKL-induced activation of NF-κB and MAPK pathways as well as induction of NFATc1 [22]. Therefore, different components of dynein complex and its regulatory molecules play diverse roles in osteoclast differentiation and function.

LIS-1, NDE1, and NDEL1 belong to an evolutionary conserved pathway that regulates microtubule dynamics and dynein functions in the cellular processes involving in neuronal and non-neuronal cell migration, mitosis, intracellular vesicular trafficking, and neuron growth cone outgrowth [19,23]. Mutations of LIS-1 and NDE1 in human or their knockout in mice cause lissencephaly, a severe brain developmental disorder characterized by brain atrophy and microcephaly with severe mental disability [23]. There have been no reported cases of human mutations in NDEL1. *Ndel1*1 knockout mice are embryonically lethal due to neuronal migration defects [24]. We have reported earlier that LIS-1 modulates trabecular bone mass during bone remodeling by regulating osteoclastogenesis and lysosome secretion through its interaction with the cytoplasmic dynein complex as well as lysosomal adaptor protein PLEKHM1 [25,26]. Nonetheless, the role of NDE1 and NDEL1 in regulation of osteoclasts *in vitro* and *in vivo* has not been elucidated.

We have previously identified that both Nde1 and Ndel11 interact with Plekhm1 in cultured murine osteoclasts, implicating an important role of these two proteins in osteoclast function [27]. To further determine the osteoclast autonomous functions of the two homologs, we carried out shRNA knockdown of *Nde*1 and generated *Ndel*1 conditional knockout mice in myeloid osteoclast precursors. The impacts of Nde1 and Ndel1 deficiency on osteoclastogenesis *in vitro* and skeletal mass *in vivo*, were evaluated.

## 2. Materials and Methods

### 2.1. Mice and Genotyping

*Ndel*1-flox (129S-*Ndel*1^tm2hr^, stock number 026958) and *Lys*M-Cre (B6.129P2-*Lyz*2^tm1(cre)Ifo^, stock number 004781) mice on 129Sv and C57BL6 background, respectively, were procured from The Jackson Laboratory (Bar Harbor, ME, USA). Homogenous floxed mice of *Ndel*1 in C57BL6 background were generated by backcrossing 129Sv mice with C57BL6 mice for greater than ten generations. Mice were genotyped for *Ndel*1-flox and *Lys*M-Cre using primers and protocol provided by The Jackson Laboratory.

### 2.2. Reagents and Antibodies

Alpha-MEM (catalog no. 78-5077EB), Alexa Fluor 488 phalloidin (catalog. A12379), Halt phosphatase inhibitor cocktail (catalog no. 78426), 10x Trypsin/EDTA (catalog. 15400-054) and Hoechst 33342 (catalog. H3570) were purchased from Thermo-Fisher Scientific (Waltham, MA, USA). High glucose DMEM (catalog no. D-5648), 3,3′-diaminobenzidine (DAB) tablets (D-5905), cOmplete EDTA-free protease inhibitor cocktail (catalog no. 4693159001), chemiluminescent detection reagents (ECL, catalog no. WBKLS0100), hydrogen peroxide (catalog no. 216763), Napthol AS-BI phosphoric acid solution (catalog no. 1802), NaK tartrate (catalog no. S6170), peroxidase-conjugated WGA (Wheat germ agglutinin) lectin (catalog no. L-7017), 10× penicillin–streptomycin-l-glutamine (PSG) (catalog no. G1146), polyvinylidene difluoride (PVDF) membrane (catalog no. IPVH00010), puromycin (Millipore Sigma), and 1 × RIPA buffer (catalog no. R0278) were obtained from Millipore Sigma (Burlington, MA, USA). Fetal bovine serum (FBS) was obtained from Hyclone (Logan, UT, USA). TransIT-LT1 transfection reagent (catalog no. MIR2300) was obtained from Mirus Bio LLC (Madison, WI, USA). All others were from Millipore Sigma.

The following antibodies were used in this study: mouse monoclonal anti-PCNA (catalog no. 2586), rabbit anti-cyclin D1 (catalog no. 2922), mouse monoclonal pan-Akt (clone 40D4, catalog no. 2920), rabbit monoclonal anti-phosphor-Akt (Ser473) (clone D9E, catalog no. 4060), rabbit anti-IκBα (catalog no. 9242), mouse monoclonal anti-phospho- IκBα (Ser32/36) (clone 5A5, catalog no. 9246), rabbit anti-ERK1/2 (catalog no. 9102), mouse monoclonal anti-phospho-ERK1/2(Thr202/Tyr204) (clone E10, catalog no. 9106), rabbit anti-SAPK/JNK (catalog no. 9252), mouse monoclonal anti-phospho-SAPK/JNK (Thr183/Tyr185) (clone G9, catalog no. 9255), horseradish peroxidase conjugated secondary antibodies against mouse (catalog no. 7074) and rabbit (catalog no. 7076) were obtained from Cell Signaling Technology (Danvers, MA, USA); mouse monoclonal anti-cathepsin K (clone 182-12G5, catalog no. MAB3324) and anti-tubulin (clone DM1A, catalog no. T9026) were obtained from Millipore Sigma (Burlington, MA, USA); mouse monoclonal against NFATc1 (catalog no. sc-7294) was purchased from Santa Cruz Biotechnology (Dallas, TX, USA).

### 2.3. Micro Computed Tomography (μCT)

Femurs were harvested from mice, cleaned of adjacent tissue, and fixed overnight in 4% paraformaldehyde in phosphate buffer saline (PBS) at 4 °C. Fixed bones were washed with 1× PBS and stored in PBS containing 0.02% sodium azide at 4 °C until assessed by μCT as described previously [28]. Briefly, the bones were loaded into a μCT40 (Scanco Medical AG, Brüttisellen, Switzerland ) equipped with scanning tube of 12.3 mm and imaged. 3D voxel was prepared by integrating the scanned images (1024 × 1024-pixel matrices for each individual planar stack) followed by Gaussian noise reduction (sigma = 0.8, support = 1). A threshold of 200 was applied at a medium resolution (E = 55 kVp, I = 145 μA, integration time = 200 ms)

### 2.4. In vitro Osteoclast Cultures

Cells from bone marrow were harvested by flushing tibias and femurs from 6–8-week-old control or conditional knockout mice and incubated in red blood cell (RBC) lysis buffer (150 mM ammonium chloride, 10 mM potassium bicarbonate, 0.1 mM EDTA, pH 7.4) for 5 min at room temperature to remove RBCs. Five million cells were plated in 100 mm petri-dish with α-10 media (α-MEM, 10% heat-inactivated FBS, 1× PSG) containing 10% CMG 14-12 (conditioned media supernatant containing recombinant M-CSF at 1 μg/mL) and grown for 4–5 days, as performed in our previous work [29]. Bone marrow monocytes (BMMs) were trypsinized and replated at 160 cells/mm^2^ in tissue culture plates/dishes with 10% CMG for monocytes or to generate mononuclear pre-osteoclasts and mature osteoclasts with 1% CMG and 100 ng/mL of recombinant RANKL for 2 and 4 days, respectively.

### 2.5. TRAP Staining

Osteoclasts cultured in 48-well tissue culture plates were fixed with 4% paraformaldehyde (PFA) in 1× PBS for 20 min at room-temperature. Cells were washed with 1× PBS twice prior to staining for TRAP with Sodium-Potassium tartrate and napthol AS-BI phosphoric acid [25]. Images were acquired with a stereomicroscope equipped with a digital camera (Discovery V12 and AxioCam; Carl Zeiss, Inc. Thornwood, NY, USA). TRAP positive osteoclasts with greater than 3 nuclei were calculated and analyzed using GraphPad Prism 9 (GraphPad Software, La Jolla, CA, USA).

### 2.6. Lentiviral Transduction for shRNA Expression

Lentiviral constructs containing the RNAi sequence 5’-ACCAACTGCAGAAATACATTA-3’ (*Nde*1-sh1) and 5’-AGTACCAGTGTGGGCGATAAA-3’ (*Nde*1-sh2) in LKO.1 were purchased from Millipore Sigma. A shRNA targeting firefly luciferase (LUC) was used as control. Lentivirus expressing shRNAs were generated by co-transfecting 293-T cells with LKO.1, ΔH8.2 (gene transfer vector) and VSVG (virus packaging vectors), using a TransIT-LT1 transfection kit (Mirus Bio LLC). Viral supernatants for each lentivirus were collected 48 h post transfection and used for transduction of BMMs in presence of M-CSF and 20 μg/mL protamine (Millipore Sigma) for 24 h and later selected with puromycin (6 μg/mL) for 3 days [30].

### 2.7. Resorption Pit Staining

Osteoclasts were cultured on bovine cortical bone slices in 48-well plates, fixed with 4% PFA in PBS for 20 min at room-temperature and washed twice with PBS for 5 min. Prior to staining the bone slices for resorption pits, bound cells were removed by brushing, followed by incubation in HRP-conjugated WGA lectin (20 μg/mL) for 60 min at room-temperature. The slices were washed with PBS twice and incubated in a solution of 0.52 mg/mL 3,3-diaminobenzidine and 0.03% hydrogen peroxide for 30 min [31]. They were later mounted on glass slides with 80% glycerol in PBS and imaged with AxioPlan2 microscope (Carl Zeiss) equipped with a digital camera (Olympus DP73, Center Valley, PA, USA).

### 2.8. Immunofluorescent Staining

Osteoclast cultures were raised on glass coverslips for 4-5 days in 24-well plates. Cells were fixed with 4% PFA for 20 min and washed twice with PBS. Cells were permeabilized and blocked in PBS containing 0.2% BSA and 0.1% Saponin (PBBS) for 30 min or permeabilized with 0.1% Triton-X100 in PBS for 10 min followed by blocking with 0.2% BSA in PBS at room temperature. Cells were probed for Lamp2 or tubulin by incubating with respective antibodies for 2 h. Target proteins were visualized with fluorescent-dye conjugated secondary antibodies in blocking buffer for 45 min in dark. F-actin and nuclei were stained with Alexa-488-conjugated phalloidin (1:300 from 1mg/mL stock) and Hoechst 33342 (1:4000 from a 10 mg/mL stock), respectively [32]. Coverslips were mounted with 80% glycerol (in PBS) post two washes with PBS and imaged using AxioImager Z1 fluorescent microscope (Carl Zeiss) containing a monochromatic (AxioCam MRm) and color (MR5c) cameras.

### 2.9. Cell Proliferation and Apoptosis Assay

BMMs cultures were raised with M-CSF for 3 days. Cell proliferation was measured using the Cell Counting kit-8 (Millipore Sigma) as per manufacturer’s instructions [33]. For detection of cell death, BMMs were cultured to raise pre-osteoclasts with M-CSF and RANKL for 3 days in a 48-well plate. Pre-osteoclasts were subjected to cytokine and serum starvation for 4 h followed by assaying for apoptosis using the Cell Death Detection ELISA^PLUS^ kit (catalog No. 11774425001, Roche, Basel, Switzerland) as per manufacturer’s instructions.

### 2.10. RNA Isolation and Real-Time Quantitative PCR

Total RNA was extracted using RNeasy mini kit (Qiagen) according to manufacturer’s instruction. First strand cDNA was synthesized using the High-Capacity cDNA Reverse Transcription kit (Thermo Fisher Scientific) from 0.5–1.0 μg of purified RNA. Quantitative real-time PCR was performed using the following primers from Thermo Fisher Scientific: *Mrps*2 (Mm00475529_m1); *Nde*1 (Mm00481033_m1) in the CFX96 Touch Real-Time PCR Detection System (Biorad Laboratories, Hercules, CA, USA) with the following cycle setup: initial denaturation at 95 °C/10 min followed by 40 cycles of denaturation (95 °C/15 s) and annealing-extension (60 °C/1 min). Relative cDNA levels were calculated using the ΔCt method by normalizing against the steadily expressing mitochondrial gene *Mrps*2.

### 2.11. Immunoblotting

Cultures of BMMs, pre-osteoclasts or osteoclasts were washed with ice-cold PBS twice prior to lysing in 1× RIPA lysis buffercontaining Complete Mini EDTA-free protease inhibitor cocktail and Halt Phosphatase inhibitor cocktail. Cell lysates were kept on ice for 30 min and then centrifuged at 14,000 rpm for 15 min at 4 °C. The clarified cell lysates were collected, total protein was estimated, and 10–30 μg was subjected to 8–12% SDS-PAGE. Resolved proteins were transferred onto a 0.45 μm PVDF membrane using the semi-dry blotting module (Biorad Laboratories). Membranes were blocked with 5% fat-free milk in Tris-buffered saline (TBS) for 1 h, followed by incubation with specific primary antibodies at 4 °C overnight. Membranes were washed and incubated with HRP conjugated secondary antibodies for 45 min. Membranes were washed thrice with TBS containing 0.1% Tween-20 (Biorad Laboratories) and used for enhanced chemiluminescent detection of target protein. Tubulin or total protein, for the respective phospho-proteins, resolved from equal amount of the same sample under identical conditions, served as control.

### 2.12. Statistics

As outlined in our previous studies and reports from others, the sample size was kept at > 6 animals per group, for α = 0.05 (two-sided) and a power of 0.95 [28,34]. Each individual mouse represented a biological replicate for each experiment, both *in vivo* as well as *in vitro,* Data in all graphs are presented as mean ± SD. A 2-tailed Student’s t-test or 1-way ANOVA with the Bonferroni procedure was used for comparison of 2 or more than 2 groups respectively. All statistical methods were performed using GraphPad Prism 9 (GraphPad Software, La Jolla, CA, USA), and a *p*-value of < 0.05 was considered significant.

## 3. Results

### 3.1. Knocking-Down Nde1 Expression in Bone Marrow Monocytes Inhibits Osteoclastogenesis In Vitro

**Figure 1:**
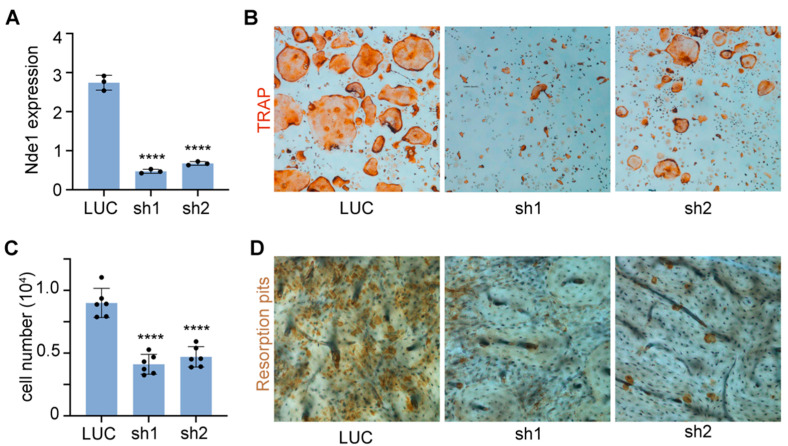
Knocking-down Nde1 expression by specific shRNAs impedes osteoclastogenesis and bone resorption in vitro. (**A**) Real-time quantitative PCR (qPCR) analysis of mRNA expression of *Nde*1 in control shRNA targeting fly luciferase (LUC) and two *Nde*1-specific shRNAs (sh1 and sh2) transduced bone marrow monocytes. Data are presented as mean + SD. *n* = 3. **** *p* < 0.0001 vs. LUC shRNA transduced control cells and analyzed by one-way ANOVA using GraphPad Prisma 9 software. (**B**) Representative images of TRAP-stained osteoclast cultures on plastic culture dishes. (**C**) The quantification of number of mature osteoclasts with more than 3-nuclei per well of a 48-well plate in (**B**). Data presented as mean + SD, *n* = 6. **** *p* < 0.0001 vs. LUC shRNA transduced cells analyzed by unpaired Student’s t-test. (**D**) The representative images of resorption pit staining.

To elucidate the role of NDE1 in osteoclast differentiation and function, we knocked down Nde1 expression by lentivirus-mediated transduction of two *Nde*1-specific shRNAs (sh1 and sh2) in wildtype bone marrow monocytes (BMMs). After a 3-day puromycin selection, the knockdown efficiency in positively transduced BMMs was validated by qPCR using *Nde*1 specific primers. As shown in Figure 1A, the mRNA level of *Nde*1 was dramatically downregulated in BMMs by both shRNAs. Unexpectedly, inhibition of Nde1 expression by sh1 and sh2 markedly reduced the number of multinucleated TRAP-positive osteoclasts compared to control shRNA targeting fly luciferase (LUC) transduced cells (Figure 1B,C). When cultured on bone slices, Nde1 knockdown cultures formed fewer resorption pits than controls (Figure 1D), probably due to decreased number of mature osteoclasts.

**Figure 2:**
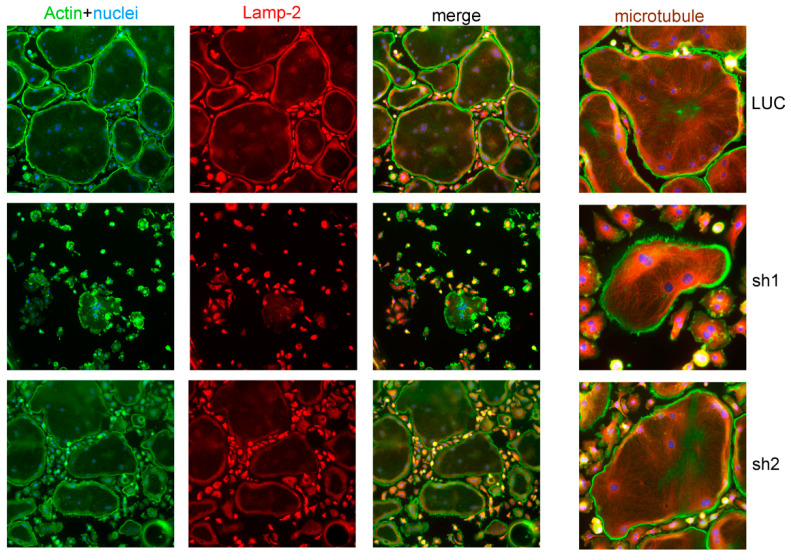
Nde1 knockdown has little impact on actin and microtubule cytoskeleton organization and lysosome distribution in osteoclasts. Bone marrow monocytes harvested from wildtype mice were transduced with shRNAs targeting LUC and *Nde1*, respectively. The positively transduced cells were cultured on glass coverslips with M-CSF and RANKL for 4 days. Cells were fixed with 4% paraformaldehyde/PBS and stained for filamentous actin (phalloidin), lysosome (Lamp-2), and microtubules (tubulin). The images are representatives of three replicate experiments.

Since Nde1 knockdown greatly attenuated osteoclastogenesis, it is difficult to assess its function in osteoclast cytoskeleton organization and lysosome transportation. In a few mature osteoclasts formed in Nde1 depletion cultures, especially *Nde*1 sh2 transduced osteoclasts, we could observe peripherally localized podosome-belt and normal pattern of microtubule fibers radiating from the nuclei towards the cell periphery revealed by actin and tubulin double staining (Figure 2). The peri-nuclear aggregation of lysosomes as seen in Plekhm1^−/−^ osteoclasts [27] was not observed in Nde1 depleting osteoclasts as demonstrated by lysosome membrane protein Lamp2 staining, suggesting that NDE1 is dispensable for lysosome transportation in osteoclasts. These results indicate that NDE1 specifically regulates osteoclastogenesis.

### 3.2. NDE1 Regulates the Proliferation and Survival of Osteoclast Precursor Cells through Modulation of Both M-CSF and RANKL Activated SIGNALING Pathways

**Figure 3:**
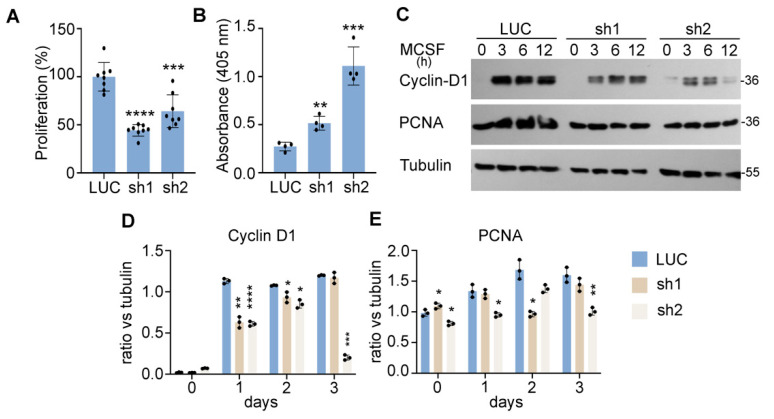
NDE1 regulates proliferation and survival of osteoclast precursor cells. (**A**) Cell proliferation was assessed by the cell count CCK-8 assay. Data are presented as mean + SD, *n* = 8, *** *p* < 0.001 and **** *p* < 0.0001 vs. LUC shRNA transduced cells analyzed by Student’s t-test. (**B**) Control and Nde1-deficient BMMs were cultured with M-CSF and RANKL for 2 days. The pre-osteoclasts were serum and cytokine starved for 3 h and apoptosis was assessed spectrophotometrically using the Cell Death ELISA kit. Data are presented as mean + SD, *n* = 5. ** *p* < 0.01 and *** *p* < 0.001 vs. LUC transduced cells analyzed by Student’s t-test. (**C**) The control and Nde1-deficient BMMs were serum-starved for overnight followed by stimulation with the 50 ng/mL of M-CSF for the indicated time. The level of cell proliferation markers, Cyclin-D1 and PCNA, was detected by immunoblotting with specific antibodies. The blotting of tubulin in the same membrane served as the loading control. (**D**,**E**) The ratio of each protein versus its loading control was estimated using ImageJ 1.53 (National Institute of Health, USA) from three independent experiments and analyzed using Graphpad Prism 9. Data presented as mean + SD, *n* = 3. * *p* < 0.05, ** *p* < 0.01, *** *p* < 0.001, **** *p* < 0.0001 vs. LUC shRNA transduced cells analyzed by unpaired Student’s *t*-test.

Optimal formation of multinucleated osteoclasts depends on the proliferation and survival of osteoclast precursor and mature cells [35]. RANKL in conjunction with M-CSF is also known to promote proliferation by stimulating DNA synthesis during the early proliferative stage of osteoclastogenesis [36]. Both cytokines play a pivotal role in survival of osteoclast lineage cells [37]. Since Nde1 knockdown resulted in marked decrease in osteoclastogenesis, we then set out to determine whether NDE1 regulates proliferation and survival of osteoclast lineage cells. To this end, we performed a cell proliferation CCK-8 assay in M-CSF-stimulated control and Nde1-knockdown BMMs. As shown in Figure 3A, Nde1 depletion by specific Nde1-targeting shRNAs caused a nearly 50% decrease in M-CSF induced proliferation of BMMs compared to control shRNA-expressing cells. In corroboration of this finding, the levels of canonical osteoclast proliferation and DNA synthesis markers cyclin-D1 and PCNA (proliferation cell nuclear antigen) was reduced in Nde1-depletion BMMs compared to control cells (Figure 3C–E). In addition, Nde1 knockdown promoted apoptosis in pre-osteoclasts as measured by a cell-death ELISA, which detects fragmented DNA in apoptotic cells (Figure 3B).

**Figure 4:**
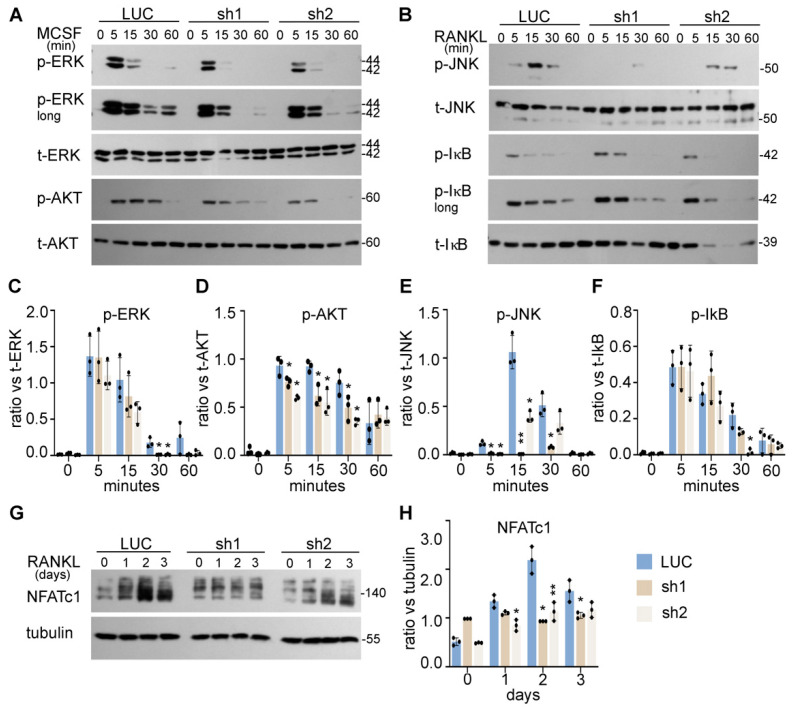
NDE1 regulates proliferation, survival, and differentiation of osteoclast precursor cells by modulating M-CSF and RANKL signaling pathways and induction of NFATc1. (**A**,**B**) The LUC, Nde1-sh1, and -sh2 transduced wildtype BMMs were serum and cytokine starved for 12 h prior to stimulation with 50 ng/mL of M-CSF or 100 ng/mL RANKL for the indicated duration, respectively. The activation of ERK and AKT induced by M-CSF and the activation of RANKL-stimulated JNK and NF-κB pathways were probed by Western blotting with phosphorylation specific antibodies. The blotting of total respective proteins served as control. (**C**–**F**) The ratio of each protein versus its total protein was estimated using ImageJ 1.53 (National Institute of Health, Bethesda, MD, USA), normalized against respective loading control from three independent experiments and analyzed using Graphpad Prism 9. Data presented as mean + SD, *n* = 3. * *p* < 0.05, ** *p* < 0.01 vs. LUC were analyzed by unpaired Student’s t-test. (**G**) The induction of NFATc1 during osteoclast differentiation was detected by western blotting using anti-NFATc1 antibody with anti-tubulin blotting as loading control. (**H**) The ratio of each protein versus its control is presented from three independent experiments as analyzed by Graphpad Prism 9. Data presented as mean + SD, *n* = 3. * *p* < 0.05 ** *p* < 0.01 vs. LUC analyzed by unpaired Student’s t-test.

The intracellular signaling pathways induced by M-CSF and RANKL coordinately regulate proliferation, survival, and differentiation of osteoclast lineage cells [8]. To determine the underlying mechanisms by which NDE1 regulates osteoclastogenesis, we examined the impacts of Nde1 suppression on M-CSF and RANKL signaling in BMMs. The control and Nde1 knockdown BMMs were serum starved overnight and stimulated with 50 ng/mL M-CSF or 100 ng/mL RANKL, respectively, for the indicated time. The activation of canonical M-CSF and RANKL signaling pathways was detected by Western blotting using specific antibodies recognizing phosphorylated signaling molecules. As evident from Figure 4A, Nde1 knockdown by two specific shRNAs dramatically inhibited the prolonged activation of ERK and AKT stimulated by M-CSF compared to control cells (Figure 4C,D). Activation of c-Jun N-terminal kinase (JNK) and NF-*κ*B pathways by RANKL plays an important role in survival of osteoclast precursors and osteoclastogenesis *in vitro* and *in vivo* [38,39]. While JNK activation peaked at 15-min post RANKL-stimulation in control cells (Figure 4B,E), this process was attenuated in Nde1-depletion cells. In contrast, RANKL-activated NF-*κ*B pathway was less affected by Nde1 knockdown as reflected by the level of phosphorylated I*κ*B (Figure 4B,F). Furthermore, the induction of osteoclast differentiation transcription factor NFATc1 upon RANKL-stimulation was decreased in NDE1-suppressed osteoclast lineage cells compared to control cells (Figure 4G,H). Taken together, these results indicate that NDE1 regulates osteoclast proliferation, survival, and differentiation by modulating M-CSF and RANKL activated signaling pathways as well as induction of NFATc1 during osteoclast differentiation.

### 3.3. NDEL1 Is Dispensable for Skeletal Homeostasis and Osteoclast Bone Resorption

After establishing a critical role for NDE1 in osteoclastogenesis, yet a definitive role for NDEL1, which shares significant structural and functional overlaps with NDE1, in osteoclastogenesis and bone resorption *in vivo* and *in vitro*, has remained elusive. As predicted by newly released Alphafold [40], both proteins contain a distinct N-terminal coiled-coil-*α*-helical domain spanning residue 10–185 and a relatively disordered C-terminal domain with one *α*-helix (residues ~240–280). Although multiple oligomeric states have been reported, homo- or hetero-dimerization of the two proteins in an extended parallel coiled-coil fashion is critical for their interactions with Dynein and LIS-1, as reported in Protein Data Bank (PDB ID: 2V71) [41,42]. The N-terminal coiled-coil and C-terminal *α*-helix are both required for dynein binding, with the former binding to the intermediate and the latter to the heavy chain of dynein, respectively [42]. LIS-1, however, binds to a dedicated region towards the C-terminal end of the coiled–coiled dimer, a site with >90% sequence identity between NDEL1 and NDE1. Germline deletion of Ndel1 in mice leads to embryonic lethality due to severe brain developmental defects [24]. To dissect how NDEL1 regulates bone modeling/remodeling and osteoclasts postnatally, we generated Ndel1 conditional knockout mice in myeloid osteoclast precursors by crossing Ndel1-floxed mice [24] with LysM-Cre mice on C57BL/6J background. The progeny of Ndel1^flox/flox;+/+^ and Ndel1^flox/flox^;LysM^Cre/Cre^ mice were used as control and Ndel1 myeloid conditional knockout (Ndel1^ΔlysM^) mice, respectively, for further *in vivo* and *in vitro* studies.

**Figure 5:**
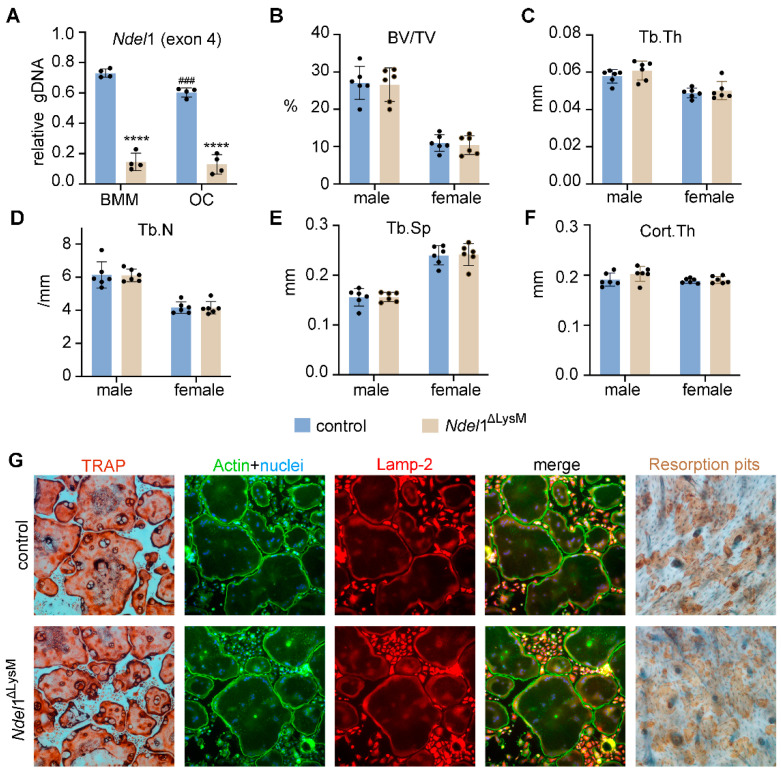
Loss of Nde1 homolog Ndel1 in myeloid osteoclast precursors is dispensable for bone homeostasis in mice and has no effect on osteoclastogenesis and bone formation in vitro. (**A**) qPCR amplification of the exon 4 of murine Ndel1 genomic DNA normalized to the transferrin receptor 1 locus using genomic DNA isolated from control and *Ndel1*^ΔLysM^ bone marrow monocytes (BMM) and osteoclasts (OC). *n* = 4. **** *p* < 0.0001 vs control; ### *p* < 0.001 vs control BMM. (**B**–**F**) μCT analyses of the trabecular and cortical compartments of distal femurs of 10-week-old control and *Ndel1*^ΔLysM^ male and female mice on C57BL/6J background. *n* = 6. (**G**) Tartarate-resistant acid phosphatase (TRAP, cultured on plastic dishes), immunofluorescent staining of actin filaments and lysosomes (cultured on glass coverslips and labeled with phalloidin and rat anti-mouse Lamp2 monoclonal antibody), and bone resorption pit (on bovine cortical bone slices) of control (top panels) and Ndel1-deficient (bottom panels) osteoclasts, respectively.

qPCR detection of the exon 4 of murine Ndel1 genomic DNA confirmed deletion of Ndel1 in osteoclast lineage cells (Figure 5A). Both male and female Ndel1^ΔlysM^ mice were born at the expected Mendelian ratio and developed normally with similar size and body weight compared to their littermate controls at 10 weeks of age. The µCT analysis of distal femurs of male and female mice exhibited similar trabecular bone volume (BV/TV), trabecular thickness (Tb.Th), trabecular number (Tb.N), trabecular spacing (Tb.Sp), and cortical thickness between control and Ndel1ΔlysM male and female mice (Figure 5B–F), indicating that loss of Ndel1 in osteoclast precursors have no impact upon bone homeostasis *in vivo*.

To enquire whether loss of Ndel1 has effect(s) on osteoclast differentiation and/or function, we cultured BMM from control and Ndel1^ΔlysM^ mice with M-CSF and RANKL *in vitro* on plastic culture dish, glass coverslips, or bovine cortical bone slices. As shown in Figure 5G, the formation of mature multinucleated TRAP^+^ osteoclasts from Ndel1^ΔlysM^ BMMs was similar to control cells. In addition, genetic loss of Ndel1 had no effects on podosome-belt formation and lysosome distribution in osteoclasts cultured on glass coverslips, with no apparent aberrations of cytoskeleton organization and lysosome transportation in Ndel1-depletion osteoclasts (Figure 5G). Furthermore, the bone resorption capacity of Ndel1-deficient and control osteoclasts cultured on bovine cortical bone slices was equivalent (Figure 5G), suggesting an intact osteoclast differentiation and function in Ndel1-deficient osteoclast lineage cells. In contrast, it has been previously reported that knockdown of Ndel1 expression in bone marrow monocytes by shRNAs *in vitro* leads to slightly decreased osteoclast formation and aberrant lysosome positioning [27]. The discrepant results obtained from Ndel1 shRNA knockdown and Ndel1 genetic deletion experiments might reflect the distinct effects of Ndel1 short-term knockdown by shRNAs and long-term genetic deletion of Ndel1 in osteoclast lineage cells due to the accrual of compensatory mechanism probably by Nde1 or Lis1 in Ndel1-null osteoclast precursor and mature cells. The exact mechanism(s) needs further investigation in future. A similar finding has been observed for transferrin receptor 1 (Tfr1) in osteoclast lineage cells. While *in vitro* knockdown of Tfr1 expression in bone marrow monocytes by shRNAs inhibits osteoclast differentiation [43], genetic deletion of Tfr1 by LysM-Cre has no effects on osteoclastogenesis [28].

**Figure 6:**
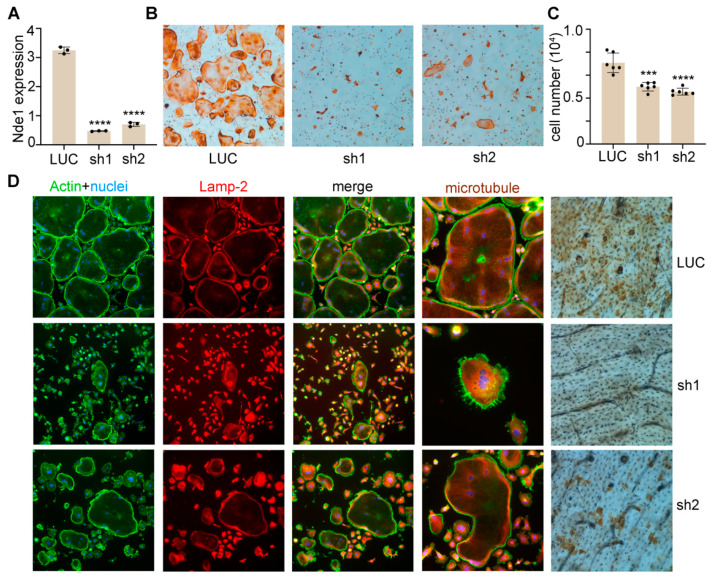
Nde1-Ndel1 double depletion has no additive effects on osteoclast differentiation, microtubule organization, lysosome distribution and function in vitro. (**A**) Real-time quantitative PCR (qPCR) analysis of mRNA expression of *Nde1* in control shRNA targeting fly luciferase (LUC) and two *Nde*1-specific shRNAs (sh1 and sh2) transduced *Ndel*1^ΔLysM^ bone marrow monocytes. Data presented as mean + SD. n = 4. **** *p* < 0.0001 vs LUC shRNA transduced control cells analyzed by one-way ANOVA using GraphPad Prisma 9 software. (**B**) Representative images of TRAP-stained osteoclast cultures on plastic culture dishes. (**C**) The quantification of number of mature osteoclasts with more than 3 nuclei per well of a 48-well plate culture in (**B**). Data presented as mean + SD, *n* = 6. *** *p* < 0.001 and **** *p* < 0.0001 vs. LUC shRNA transduced cells analyzed by unpaired Student’s t-test. (**D**) Control and Nde1 knockdown cells were cultured on glass coverslips or bone slices with M-CSF and RANKL for 4 days. Cells were fixed with 4% paraformaldehyde/PBS and stained for filamentous actin (phalloidin), lysosome (Lamp-2), and microtubules (tubulin) on glass or for resorption pits on bone slices. The images are representatives of three replicate experiments.

Extensive structural and functional overlaps between NDEL1 and NDE1 allowed us to next test whether Nde1 compensates for the deletion of Ndel1 in osteoclasts. The mRNA level of Nde1 was slightly but significantly increased in Ndel1-deficient BMMs relative to control cells (Figure 1A LUC vs. Figure 6A LUC, *p* < 0.01 by Student’s t-test). We knocked down Nde1 expression in Ndel1^ΔLysM^ BMMs as done for WT BMMs earlier. The knockdown efficiency was validated by qPCR (Figure 6A) and osteoclast formation and activity were measured. Nde1 depletion significantly reduced the number of multinucleated TRAP-positive osteoclasts in compared to LUC transduced Ndel1^ΔLysM^ control (Figure 6B,C). Like our earlier observation (Figure 2), a few mature osteoclasts which formed in Nde1/Ndel1 double depletion cultures, especially Nde1 sh2 transduced osteoclasts, had an intact cytoskeleton and lysosome distribution, similar to our finding with Nde1 single knockdown cultures. It is likely that Lis-1 compensates for the loss of Nde1 and Ndel1, since we have previously identified that it can bind to Plekhm1 and regulate dynein function in osteoclasts [26,27]. Finally, Nde1 knockdown had a pronounced reduction in resorption pits compared to Ndel1^ΔLysM^ osteoclast (Figure 6D). Interestingly though, the Nde1-Ndel1 double deletion had no additive effect on the phenotype, suggesting a predominant role of NDE1 amongst this ortholog pair in osteoclastogenesis.

## 4. Discussion

LIS-1, NDE1, and NDEL1 form an evolutionary conserved pathway that regulates microtubule dynamics and the cytoplasmic dynein from yeast to mammalian cells [19]. Mutations of LIS-1 and NDE1 in humans cause the brain disorder of lissencephaly and mental illness [44,45,46]. Germline-deletion of each of these proteins in mice causes embryonic lethality due to the severe neurodevelopmental defects [24]. We have previously identified that all these three proteins interact with the lysosomal adaptor protein PLEKHM1 indicating that they may function in lysosome transportation and secretion during osteoclast bone resorption [27]. The conditional deletion of LIS-1 in myeloid osteoclast precursors induced by *Lys*M-cre attenuates osteoclastogenesis and leads to increased trabecular bone mass in mice [25]. In this study, we have demonstrated that loss of *Nde*l1 in *Lys*M-Cre expressing myeloid lineage cells has little effects on osteoclastogenesis, bone resorption, and is dispensable for bone homeostasis. This finding is unexpected since NDEL1 have been previously reported to be crucial for lysosomal and vesicular transport along the microtubule network [24,47].

Although distinct functions between NDE1 and NDEL1 have been reported in neurons [23,24], both proteins share significant structural similarities and functional overlaps [48]. We, therefore, postulate that NDEL1 in osteoclasts may be functionally redundant with NDE1. To our surprise, depletion of Nde1 expression by specific shRNAs leads to the similar aberration of osteoclastogenesis in control and *NdeL*1^Δ*Lys*M^ BMMs, suggesting that NDE1 plays a unique and distinct role from NDEL1 in regulation of osteoclast formation. Although both LIS-1 and NDE1 regulate osteoclastogenesis through modulation of M-CSF and RANKL signaling pathways, the underlying mechanisms are quite different. Loss of Lis-1 in BMMs results in the prolonged JNK activation upon RANKL stimulation, which induces apoptosis in osteoclast precursor cells with minimum effect on cell proliferation [25,26], whereas down-regulation of Nde1 in BMMs inhibits the M-CSF-induced AKT and RANKL-stimulated transient JNK activation, leading to decreased cell proliferation, survival, and NFATc1 induction.

It is still unknown how NDE1 regulates M-CSF and RANKL signaling in osteoclasts. It is likely mediated by one of its interacting partners from a component of the dynein complex, the 8 kDa dynein light chain (LC8). The LC8 is an integral protein of the functional dynein motor complex. In addition to its essential role in dynein-mediated motor activity, LC8 has been reported to inhibit osteoclast differentiation by regulating RANKL downstream pathways [22]. Notably, NDE1, but not NDEL1, interacts with LC8 via a distinct 4-residue binding site (200–203) located upstream of the C-terminal α-helix [49]. It is therefore likely that, NDE1-LC8 interaction plays a crucial role in osteoclasts, with reduction of NDE1 expression causing LC8 mediated inhibition of osteoclast proliferation. This mechanism will be further investigated in the future using *Nde*1-null BMMs and loss-of- and gain-of-function of LC8 mutants in osteoclast lineage cells. Yet another mechanistic possibility of NDE1 mediated osteoclast regulation is via modulating precursor density. Notably, NDE1 plays a critical role in mitosis, regulating chromosomal distribution and progression [19,23,42,45]. We and others have previously reported a critical role of precursor cell density in the formation of mature osteoclasts [25,35]. We observed a significant reduction in cell proliferation and number (Figure 1C, Figure 3A and Figure 6C) upon Nde1 knockdown, suggesting that the reduction in cell number could have ablated osteoclast formation. The precise mechanistic underpinnings on how NDE1 regulates mitosis in osteoclast lineage cells warrants further investigation.

In summary, we have provided evidence supporting a novel role of NDE1 in osteoclasts. While NDEL1 is dispensable for osteoclast formation and activity, NDE1 regulates osteoclast precursor proliferation and subsequent formation of mature osteoclasts by modulating M-CSF and RANKL activated pathways and induction of NFATc1.

## Data Availability

Available upon request.

## References

[1] Zaidi M. (2007). Skeletal remodeling in health and disease. Nat. Med..

[2] Raggatt L.J., Partridge N.C. (2010). Cellular and Molecular Mechanisms of Bone Remodeling. J. Biol. Chem..

[3] Novack D.V., Teitelbaum S.L. (2008). The osteoclast: Friend or foe?. Annu. Rev. Pathol. Mech. Dis..

[4] Walker D.G. (1975). Control of bone resorption by hematopoietic tissue. The induction and reversal of congenital osteopetrosis in mice through use of bone marrow and splenic transplants. J. Exp. Med..

[5] Xiao Y., Palomero J., Grabowska J., Wang L., de Rink I., van Helvert L., Borst J. (2017). Macrophages and osteoclasts stem from a bipotent progenitor downstream of a macrophage/osteoclast/dendritic cell progenitor. Blood Adv..

[6] Boyle W.J., Simonet W.S., Lacey D.L. (2003). Osteoclast differentiation and activation. Nature.

[7] Nakashima T., Hayashi M., Takayanagi H. (2012). New insights into osteoclastogenic signaling mechanisms. Trends Endocrinol. Metab..

[8] Teitelbaum S.L., Ross F.P. (2003). Genetic regulation of osteoclast development and function. Nat. Rev. Genet..

[9] Takayanagi H., Kim S., Koga T., Nishina H., Isshiki M., Yoshida H., Saiura A., Isobe M., Yokochi T., Inoue J.-I. (2002). Induction and Activation of the Transcription Factor NFATc1 (NFAT2) Integrate RANKL Signaling in Terminal Differentiation of Osteoclasts. Dev. Cell.

[10] Teitelbaum S.L. (2011). The osteoclast and its unique cytoskeleton. Ann. N. Y. Acad. Sci..

[11] Zhao H. (2012). Membrane Trafficking in Osteoblasts and Osteoclasts: New Avenues for Understanding and Treating Skeletal Diseases. Traffic.

[12] Destaing O., Saltel F., Géminard J.-C., Jurdic P., Bard F. (2003). Podosomes Display Actin Turnover and Dynamic Self-Organization in Osteoclasts Expressing Actin-Green Fluorescent Protein. Mol. Biol. Cell.

[13] Luxenburg C., Geblinger D., Klein E., Anderson K., Hanein D., Geiger B., Addadi L. (2007). The Architecture of the Adhesive Apparatus of Cultured Osteoclasts: From Podosome Formation to Sealing Zone Assembly. PLoS ONE.

[14] Väänänen H.K., Horton M. (1995). The osteoclast clear zone is a specialized cell-extracellular matrix adhesion structure. J. Cell Sci..

[15] Destaing O., Saltel F., Gilquin B., Chabadel A., Khochbin S., Ory S., Jurdic P. (2005). A novel Rho-mDia2-HDAC6 pathway controls podosome patterning through microtubule acetylation in osteoclasts. J. Cell Sci..

[16] Blangy A., Bompard G., Guerit D., Marie P., Maurin J., Morel A., Vives V. (2020). The osteoclast cytoskeleton—Current understanding and therapeutic perspectives for osteoporosis. J. Cell Sci..

[17] Ross J.L., Shuman H., Holzbaur E.L., Goldman Y.E. (2008). Kinesin and Dynein-Dynactin at Intersecting Microtubules: Motor Density Affects Dynein Function. Biophys. J..

[18] Pfister K.K., Shah P.R., Hummerich H., Russ A., Cotton J., Annuar A.A., King S.M., Fisher E.M.C. (2006). Genetic Analysis of the Cytoplasmic Dynein Subunit Families. PLoS Genet..

[19] Lam C., Vergnolle M.A.S., Thorpe L., Woodman P.G., Allan V. (2010). Functional interplay between LIS1, NDE1 and NDEL1 in dynein-dependent organelle positioning. J. Cell Sci..

[20] Ng P.Y., Cheng T.S., Zhao H., Ye S., Ang E.S., Khor E.C., Feng H.-T., Xu J., Zheng M.H., Pavlos N.J. (2013). Disruption of the dynein-dynactin complex unveils motor-specific functions in osteoclast formation and bone resorption. J. Bone Miner. Res..

[21] Pavlos N.J., Cheng T.S., Qin A., Ng P.Y., Feng H.-T., Ang E.S.M., Carrello A., Sung C.-H., Jahn R., Zheng M.-H. (2011). Tctex-1, a Novel Interaction Partner of Rab3D, Is Required for Osteoclastic Bone Resorption. Mol. Cell. Biol..

[22] Kim H., Hyeon S., Kim H., Yang Y., Huh J.Y., Park D.R., Lee H., Seo D.-H., Kim H.-S., Lee S.Y. (2013). Dynein Light Chain LC8 Inhibits Osteoclast Differentiation and Prevents Bone Loss in Mice. J. Immunol..

[23] Bradshaw N.J., Hayashi M.A.F. (2017). NDE1 and NDEL1 from genes to (mal)functions: Parallel but distinct roles impacting on neurodevelopmental disorders and psychiatric illness. Cell. Mol. Life Sci..

[24] Sasaki S., Mori D., Toyo-Oka K., Chen A., Garrett-Beal L., Muramatsu M., Miyagawa S., Hiraiwa N., Yoshiki A., Wynshaw-Boris A. (2005). Complete Loss of Ndel1 Results in Neuronal Migration Defects and Early Embryonic Lethality. Mol. Cell. Biol..

[25] Ye S., Fujiwara T., Zhou J., Varughese K.I., Zhao H. (2016). LIS1 Regulates Osteoclastogenesis through Modulation of M-SCF and RANKL Signaling Pathways and CDC42. Int. J. Biol. Sci..

[26] Ye S., Fowler T.W., Pavlos N.J., Ng P.Y., Liang K., Feng Y., Zheng M., Kurten R., Manolagas S.C., Zhao H. (2011). LIS1 Regulates Osteoclast Formation and Function through Its Interactions with Dynein/Dynactin and Plekhm1. PLoS ONE.

[27] Fujiwara T., Ye S., Castro-Gomes T., Winchell C.G., Andrews N.W., Voth D.E., Varughese K.I., Mackintosh S.G., Feng Y., Pavlos N. (2016). PLEKHM1/DEF8/RAB7 complex regulates lysosome positioning and bone homeostasis. JCI Insight.

[28] Das B.K., Wang L., Fujiwara T., Zhou J., Aykin-Burns N., Krager K.J., Lan R., Mackintosh S.G., Edmondson R., Jennings M.L. (2021). Transferrin Receptor 1-Mediated Iron Uptake Regulates Bone Mass in Mice via Osteoclast Mitochondria and Cytoskeleton. bioRxiv.

[29] Fujiwara T., Zhou J., Ye S., Zhao H. (2016). RNA-binding protein Musashi2 induced by RANKL is critical for osteoclast survival. Cell Death Dis..

[30] Zhou J., Fujiwara T., Ye S., Li X., Zhao H. (2015). Ubiquitin E3 Ligase LNX2 is Critical for Osteoclastogenesis *in vitro* by Regulating M-CSF/RANKL Signaling and Notch2. Calcif. Tissue Int..

[31] Zhao H., Laitala-Leinonen T., Parikka V., Väänänen H.K. (2001). Downregulation of Small GTPase Rab7 Impairs Osteoclast Polarization and Bone Resorption. J. Biol. Chem..

[32] Zhao H., Väänänen H.K. (2006). Pharmacological Sequestration of Intracellular Cholesterol in Late Endosomes Disrupts Ruffled Border Formation in Osteoclasts. J. Bone Miner. Res..

[33] Das B., Kannan A., Nguyen Q., Gogoi J., Zhao H., Gao L. (2021). Selective Inhibition of Aurora Kinase A by AK-01/LY3295668 Attenuates MCC Tumor Growth by Inducing MCC Cell Cycle Arrest and Apoptosis. Cancers.

[34] Wang L., Fang B., Fujiwara T., Krager K., Gorantla A., Li C., Feng J.Q., Jennings M.L., Zhou J., Aykin-Burns N. (2018). Deletion of ferroportin in murine myeloid cells increases iron accumulation and stimulates osteoclastogenesis *in vitro* and in vivo. J. Biol. Chem..

[35] Rahman M.M., Takeshita S., Matsuoka K., Kaneko K., Naoe Y., Sakaue-Sawano A., Miyawaki A., Ikeda K. (2015). Proliferation-coupled osteoclast differentiation by RANKL: Cell density as a determinant of osteoclast formation. Bone.

[36] Zhou P., Kitaura H., Teitelbaum S., Krystal G., Ross F.P., Takeshita S. (2006). SHIP1 negatively regulates proliferation of osteoclast precursors via Akt-dependent alterations in D-type cyclins and p27. J. Immunol..

[37] Xu F., Teitelbaum S. (2013). Osteoclasts: New Insights. Bone Res..

[38] Franzoso G., Carlson L., Xing L., Poljak L., Shores E.W., Brown K.D., Leonardi A., Tran T., Boyce B.F., Siebenlist U. (1997). Requirement for NF-κB in osteoclast and B-cell development. Genes Dev..

[39] Ikeda F., Nishimura R., Matsubara T., Tanaka S., Inoue J.-I., Reddy S.V., Hata K., Yamashita K., Hiraga T., Watanabe T. (2004). Critical roles of c-Jun signaling in regulation of NFAT family and RANKL-regulated osteoclast differentiation. J. Clin. Investig..

[40] Jumper J., Evans R., Pritzel A., Green T., Figurnov M., Ronneberger O., Tunyasuvunakool K., Bates R., Žídek A., Potapenko A. (2021). Highly accurate protein structure prediction with AlphaFold. Nature.

[41] Derewenda U., Tarricone C., Choi W.C., Cooper D.R., Lukasik S., Perrina F., Tripathy A., Kim M.H., Cafiso D.S., Musacchio A. (2007). The Structure of the Coiled-Coil Domain of Ndel1 and the Basis of Its Interaction with Lis1, the Causal Protein of Miller-Dieker Lissencephaly. Structure.

[42] Soares D.C., Bradshaw N.J., Zou J., Kennaway C.K., Hamilton R.S., Chen Z.A., Wear M.A., Blackburn E.A., Bramham J., Böttcher B. (2012). The Mitosis and Neurodevelopment Proteins NDE1 and NDEL1 Form Dimers, Tetramers, and Polymers with a Folded Back Structure in Solution. J. Biol. Chem..

[43] Ishii K.-A., Fumoto T., Iwai K., Takeshita S., Ito M., Shimohata N., Aburatani H., Taketani S., Lelliott C.J., Vidal-Puig A. (2009). Coordination of PGC-1β and iron uptake in mitochondrial biogenesis and osteoclast activation. Nat. Med..

[44] Pilz D.T., Matsumoto N., Minnerath S., Mills P., Gleeson J.G., Allen K.M., Walsh C.A., Barkovich A.J., Dobyns W.B., Ledbetter D.H. (1998). LIS1 and XLIS (DCX) mutations cause most classical lissencephaly, but different patterns of malformation. Hum. Mol. Genet..

[45] Feng Y., Walsh C. (2004). Mitotic Spindle Regulation by Nde1 Controls Cerebral Cortical Size. Neuron.

[46] Shu T., Ayala R., Nguyen M.-D., Xie Z., Gleeson J.G., Tsai L.-H. (2004). Ndel1 Operates in a Common Pathway with LIS1 and Cytoplasmic Dynein to Regulate Cortical Neuronal Positioning. Neuron.

[47] Liang Y., Yu W., Li Y., Yang Z., Yan X., Huang Q., Zhu X. (2004). Nudel functions in membrane traffic mainly through association with Lis1 and cytoplasmic dynein. J. Cell Biol..

[48] Bradshaw N.J., Hennah W., Soares D.C. (2013). NDE1 and NDEL1: Twin neurodevelopmental proteins with similar “nature” but different “nurture”. Biomol. Concepts.

[49] McKenney R.J., Weil S.J., Scherer J., Vallee R.B. (2011). Mutually Exclusive Cytoplasmic Dynein Regulation by NudE-Lis1 and Dynactin. J. Biol. Chem..

